# Color-to-Grayscale: Does the Method Matter in Image Recognition?

**DOI:** 10.1371/journal.pone.0029740

**Published:** 2012-01-10

**Authors:** Christopher Kanan, Garrison W. Cottrell

**Affiliations:** Department of Computer Science and Engineering, University of California San Diego, La Jolla, California, United States of America; Tel Aviv University, Israel

## Abstract

In image recognition it is often assumed the method used to convert color images to grayscale has little impact on recognition performance. We compare thirteen different grayscale algorithms with four types of image descriptors and demonstrate that this assumption is wrong: not all color-to-grayscale algorithms work equally well, even when using descriptors that are robust to changes in illumination. These methods are tested using a modern descriptor-based image recognition framework, on face, object, and texture datasets, with relatively few training instances. We identify a simple method that generally works best for face and object recognition, and two that work well for recognizing textures.

## Introduction

Modern descriptor-based image recognition systems often operate on grayscale images, with little being said of the mechanism used to convert from color-to-grayscale. This is because most researchers assume that the color-to-grayscale method is of little consequence when using robust descriptors. However, since many methods for converting to grayscale have been employed in computer vision, we believe it is prudent to assess whether this assumption is warranted. The most common techniques are based on weighted means of the red, green, and blue image channels (e.g., *Intensity* and *Luminance*), but some methods adopt alternative strategies to generate a more perceptually accurate representation (e.g., *Luma* and *Lightness* [1]) or to preserve subjectively appealing color contrast information in grayscale images (e.g., *Decolorize* [2]). A priori, none of these criteria suggest superior recognition performance.

The main reason why grayscale representations are often used for extracting descriptors instead of operating on color images directly is that grayscale simplifies the algorithm and reduces computational requirements. Indeed, color may be of limited benefit in many applications and introducing unnecessary information could increase the amount of training data required to achieve good performance.

In this paper we compare thirteen different methods for converting from color-to-grayscale. While we do not evaluate every method that has been developed, we evaluate all of the widely used methods, as well as some less well known techniques (e.g., *Decolorize*). All of the methods are computationally inexpensive, i.e., they all have linear time complexity in the number of pixels. This comparison is performed using the Naive Bayes Nearest Neighbor (NBNN) [3] image recognition framework and four different types of image descriptors. Our objective is to determine if the grayscale representation used significantly influences performance and if so, to identify which method is preferred regardless of the dataset or descriptor type.

Our experiments are conducted with relatively few instances, since classifier performance is much more sensitive to the quality of the descriptors in this setting [4]. One reason for this phenomenon is that an image recognition system can obtain invariance properties simply by training it with more data, as long as the additional data exhibits the same variation as the test set [5]. For many applications this is infeasible (e.g., automated surveillance systems for detecting suspected criminals) and it could reduce execution speed for some non-parametric classification algorithms, e.g., nearest neighbor. If a descriptor is not suitably robust when the size of the training set is small, the classifier may inappropriately separate the categories. We believe this is especially likely with large changes in illumination.

Related work has shown that illumination conditions and camera parameters can greatly influence the properties of several recent image descriptor types [6]. This suggests grayscale algorithms that are less sensitive to illumination conditions fmay exhibit superior performance when illumination is variable. To our knowledge, this is the first time color-to-grayscale algorithms have been evaluated in a modern descriptor-based image recognition framework on established benchmark datasets.

## Methods

### Color-to-Grayscale Algorithms

**Figure 1:**
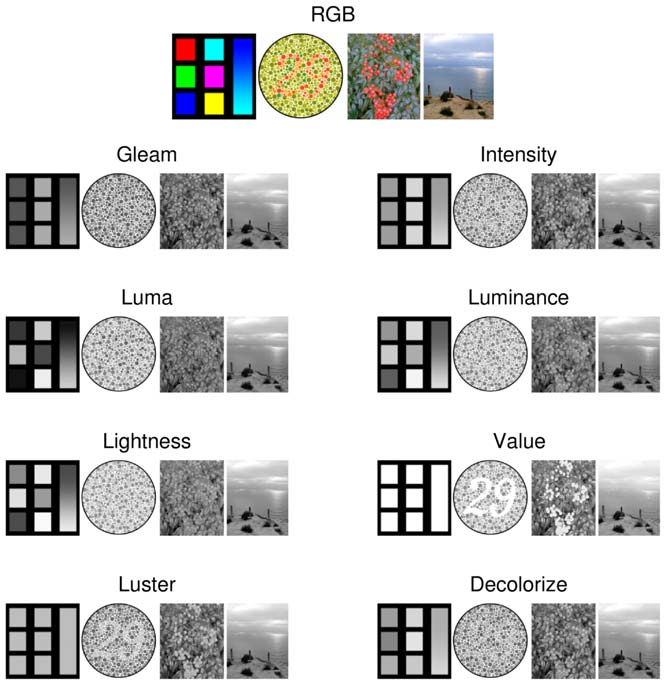
Qualitative comparison of color-to-grayscale algorithms. The four images shown are: (1) a panel of fully saturated colors; (2) Ishihara plate 3, in which a person with normal vision will see the number 29, while a person with red-green deficient vision may see the number 70; (3) a green shrub laden with red berries; and (4) a picture of the Pacific Ocean. All images are shown gamma corrected so that the details are not excessively dark, except for *Gleam*, *Luma*, and *Lightness*. The color panel contains fully saturated colors, which *Value*, *Intensity*, and *Luster* convert to the same shade of gray; however, humans do not perceive these colors as having equivalent brightness which is a trait captured by *Lightness* and *Luminance*. *Gleam*, *Intensity*, *Luminance*, *Lightness*, and *Decolorize* all lose most of the chromatic contrast present in the Ishihara plate, while *Luster*, and *Value* preserve it. The same pattern of chromatic contrast degradation is present in the fruit image, with the fruit becoming much more difficult to distinguish from the leaves for some of the methods.

In this section we briefly describe thirteen methods with linear time complexity for converting from color-to-grayscale, i.e., functions 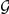 that take a 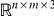 color image and convert it to a 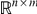 representation. All image values are assumed to be between 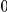 and 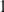. Let 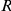, 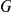, and 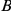 represent linear (i.e., not gamma corrected) red, green, and blue channels. The output of each grayscale algorithm is between 0 and 1. Since some methods have names like *Luster* and *Luminance*, we denote all grayscale algorithms by capitalizing the first letter and italicizing in the text. All transformations are applied component-wise, i.e., applied independently to each pixel. Several of the methods use the standard gamma correction function 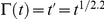 [7]. We denote gamma corrected channels as 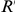, 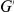, and 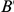. The output of the grayscale algorithms on several images is shown in Fig. 1.

Perhaps the simplest color-to-grayscale algorithm is *Intensity* [1]. It is the mean of the RGB channels:
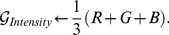
(1)Although *Intensity* is calculated using linear channels, in practice gamma correction is often left intact when using datasets containing gamma corrected images. We call this method *Gleam*:
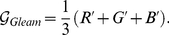
(2)In terms of pixel values, *Intensity* and *Gleam* produce very different results. Since 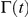 is a concave function, Jensen's inequality [7] implies that *Gleam* will never produce a representation with values greater than gamma corrected *Intensity*, and it follows that
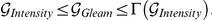
When gamma corrected *Intensity* and *Gleam* are both applied to natural images, we found that *Gleam* produces pixel values around 20–25% smaller on average.

Unlike *Intensity* and *Gleam*, *Luminance* [8] is designed to match human brightness perception by using a weighted combination of the RGB channels:
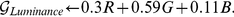
(3)
*Luminance* does not try to match the logarithmic nature of human brightness perception, but this is achieved to an extent with subsequent gamma correction. *Luminance* is the standard algorithm used by image processing software (e.g., GIMP). It is implemented by MATLAB's “rgb2gray” function, and it is frequently used in computer vision (e.g. [9]). *Luma* is a similar gamma corrected form used in high-definition televisions (HDTVs) [1]:
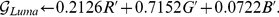
(4)


*Lightness* is a perceptually uniform grayscale representation used in the CIELAB and CIELUV color spaces [10]. This means an increment in *Lightness* should more closely correspond to human perception, which is achieved via a nonlinear transformation of the RGB color space [10],
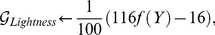
(5)where 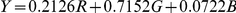, and
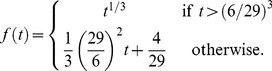
(6)We have normalized *Lightness* to range from 0 to 1, instead of the usual range of 0 to 100. The *Lightness* nonlinearity 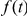 implements a form of gamma correction.

*Value* is the achromatic channel in the Hue, Saturation, and Value (HSV) color space and it provides absolute brightness information. It is computed by taking the maximum of the RGB channels [10]:
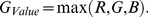
(7)Since gamma correction is a monotonically increasing function it follows that,
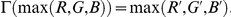
HSV is occasionally used in image recognition (e.g., [9], [11], [12]), but *Value* is equally sensitive to changes in the brightness of one color channel as it is to changes to all color channels, so we expect it to perform poorly when significant brightness variation is present.

*Luster* is the L channel in the HLS (Hue, Lightness, and Saturation) color space [1]. We changed its name from lightness to *Luster* so it is not confused with CIELAB's *Lightness* channel. *Luster* is the mean of the minimum and maximum RGB values, i.e.,
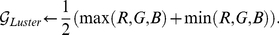
(8)It is less sensitive to changes in brightness than *Value* since any fully saturated primary color will maximize *Value*, but all three channels must be fully saturated to maximize *Luster*. Both HLS and HSV were designed to be more easily manipulated when designing computer graphics compared to RGB color space by decoupling color and brightness, rather than attempting to mimic human perception or to achieve brightness invariance.

*Decolorize* [2] is designed to preserve and enhance color contrast when converting to grayscale. There are a few algorithms designed with the same intent, but unlike others *Decolorize* has linear time complexity in the number of pixels. Cadik [13] had 119 subjects subjectively evaluate images processed using *Decolorize*, and the subjects gave it the highest overall score compared to six other methods. Qualitatively, *Decolorize* preserves color contrast in natural images moderately well; however, it does not discriminate between classification relevant and irrelevant details. The algorithm begins by converting to the YPQ color space, where the Y channel is almost identical to *Luminance*, and then it expresses the grayscale image as a piecewise linear mapping of these channels and their saturation. The algorithm is somewhat complex, so we do not provide implementation details.

We also evaluate gamma corrected forms of *Intensity*, *Luminance*, *Value*, *Luster*, and *Decolorize*, which are denoted *Intensity* 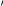, *Luminance* 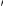, *Value* 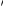, *Luster* 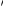, and *Decolorize* 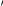, respectively. In all cases the standard gamma correction function 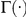 is used. This is not performed for *Gleam*, *Luma*, and *Lightness* since they have forms of gamma correction built into them.

### Image Descriptors

Our experiments are performed using four descriptor types: SIFT [14], SURF [15], Geometric Blur [16], and Local Binary Patterns (LBP) [17]. Our objective is not to determine which descriptor works best, but to see if the method of converting from color-to-grayscale is consistent across descriptor types. Each of these local descriptors are extracted from multiple spatial locations in the image, and this spatial information is used by the image recognition framework, as described in the next section. Before extracting descriptors, each image is resized to make its smallest dimension 128 with the other dimension resized accordingly to preserve the image's aspect ratio. We choose standard settings for each descriptor type.

SIFT is a popular feature descriptor that is robust to changes in illumination [14]. SIFT descriptors are computed from gradient orientation histograms weighted by the gradient magnitude computed over local neighborhoods. We densely extract 128-dimensional descriptors using 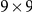 pixel spatial bins with a sampling density of 5 pixels. We use the dense SIFT implementation provided in the VLFeat toolbox [18]. About 500 descriptors are produced per image.

SURF [15] is a rotation invariant descriptor inspired by SIFT, but it uses Haar wavelets instead of the image's gradient to quickly identify interest points and generate features. The features at an interest point are the sum of the Haar wavelet responses. We use the OpenSURF implementation [19], with five octaves, a hessian threshold of 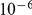, and an “extended” 128-dimensional representation. SURF produces about 100 descriptors per image.

Geometric Blur (GB) [16] descriptors are extracted by applying a spatially varying blur to oriented edge channels, with the amount of blur increasing from the center of each descriptor. Like SIFT, GB descriptors contain neighborhood information. We use standard parameters, i.e., the descriptors are computed at 300 randomly sampled points with 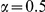 and 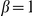. The algorithm produces 300 204-dimensional descriptors per image. See [16] for additional details.

LBP [17] descriptors have been used for texture and face recognition. Unlike the other descriptors we use, they do not directly operate on an image's gradient or edge-like features. Instead the image's pixels are used to create a local histogram of “binary patterns,” which are quantized into a 58-dimensional feature vector. We use the VLFeat [18] implementation of LBP with a cell size of 12 pixels, and we compute LBP descriptors at 3 image scales (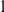, 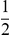, and 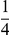), which are concatenated together to form a 174-dimensional representation. About 150 descriptors are produced per image. LBP is locally invariant to monotonically increasing changes in brightness.

### Image Recognition Framework

The Naive Bayes Nearest Neighbor (NBNN) framework [3] relies solely on the discriminative ability of the individual descriptors, making it an excellent choice for evaluating color-to-grayscale algorithms. NBNN assumes each descriptor is statistically independent (i.e., the Naive Bayes assumption). Given a new image 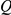 with descriptors 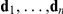, the distance to each descriptor's nearest neighbor is computed for each category 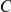. These distances are summed for each category and the one with the smallest total is chosen. Assuming that all training images have their descriptors extracted and stored, NBNN is summarized as:

1. Compute descriptors 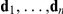 for an image 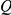.
2. For each 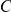, compute the nearest neighbor of every 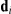 in 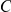: 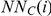.
3. Classify: 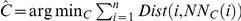.

As in [3], 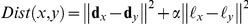, where 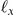 is the normalized location of descriptor 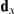, 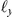 is the normalized location of descriptor 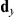, and 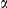 modulates the influence of descriptor location. We use 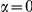 for the Barnard et al. dataset, described below, since it exhibits substantial rotation variation. We use 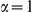 for the other datasets.

Using 15 training instances per category for Caltech 101 with SIFT descriptors, Boiman et al. [3] reported achieving 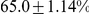 accuracy while our *Intensity* 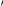 results with SIFT are 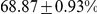. Boiman et al. did not report which grayscale method they used.

## Results

**Figure 2:**
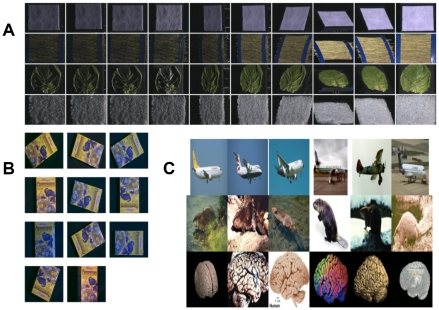
Example dataset images. (**A**): Images from four CUReT categories: Felt, Straw, Lettuce, and Salt Crystals. (**B**): The “Crucheroos” object from Barnard et al. observed in all illumination conditions. (**C**): Six sample images for three Caltech 101 categories. Images from AR are not shown to comply with PLoS privacy guidelines.

We perform recognition experiments in three domains: (1) faces (AR Face Dataset [20]), textures (CUReT [21]), and objects (Barnard et al. [22] and Caltech 101 [23]). Example images from all datasets except AR are shown in Fig. 2. Images from AR are not shown to comply with PLoS privacy guidelines.

After computing training descriptors, we subtract their mean. This is followed by applying zero-phase whitening (ZCA) for AR, CUReT, and Barnard et al. datasets to induce isotropic covariance [24]. For Caltech 101, principal component analysis whitening is used instead to reduce the dimensionality to 80, retaining at least 85% of the variance for all descriptor types. This number was chosen based on the memory and speed limitations imposed by NBNN, and when this was done for the other datasets it had negligible impact on the relative performance of the best algorithms compared to using ZCA whitening. Finally, the descriptors are normalized to unit length. These same steps are applied to descriptors during testing.

We perform 30-fold cross-validation per dataset for each color-to-grayscale method with the same train and test partitions used in each combination. For each cross-validation run we calculate the mean per-class accuracy, the standard method for Caltech 101 [23]. For each dataset and descriptor combination we provide dot plots of the mean per class accuracy. The dot plots are sorted by the mean performance across descriptors. Dot plots provide a more compact representation than bar charts and allow for easy comparison of the methods.

We use multiple comparison tests (Wilcoxon signed-rank tests) to determine which color-to-grayscale algorithms are significantly different from the method with the greatest mean performance for each descriptor type. Holm-Bonferroni correction is used to ensure that the overall Type I (false positive) error rate is 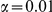. In the dot plots we indicate which methods are not statistically different from the best method for each descriptor type with a yellow triangle.

**Figure 3:**
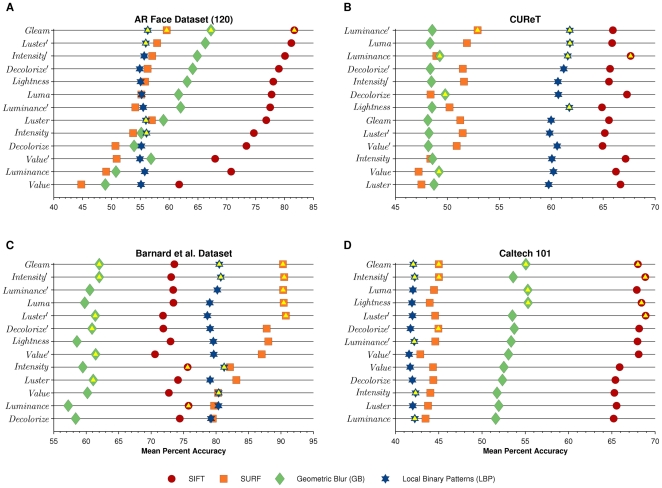
Results for each dataset. Methods that are not statistically different from the method with the greatest mean performance within each descriptor type are indicated with a gold triangle. The 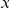-axis is the mean per-class accuracy. Each dot plot is sorted by the mean accuracy across descriptors, so that the best grayscale method across methods will be near the top of each dot plot. See text for detailed analysis. (**A**): Performance of each descriptor type on the AR Face dataset. (**B**): Performance of each descriptor type on CUReT. (**C**): Performance of each descriptor type on the Barnard et al. dataset. (**D**): Performance of each descriptor type on Caltech 101.

Results for all four datasets are shown in Fig. 3, and they are described in detail below. We focus our analysis on the methods that are top performers for multiple descriptor types.

### AR Face Dataset

The Aleix and Robert (AR) dataset [20] is a large face dataset containing over 4,000 color face images under varying lighting, expression, and disguise conditions. In our experiments we omit images with disguises and changes in expression, leaving eight neutral facial expression images per person (see [20] for example images). In each cross-validation run, we randomly choose one training image per person and six testing images. Because there are large changes in brightness, methods that are not robust to these changes could dramatically impair performance. Chance performance is 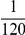.

Our results on the AR dataset are provided in Fig. 3A. For SIFT, SURF, and GB, there is a large performance gap between the best and worst methods, consistent with our hypothesis that the choice of grayscale algorithm is especially important when the number of training instances is small and there is a large amount of brightness variation. *Gleam* performs well for all four descriptors. *Value* performs poorly for all descriptors. SIFT performs best compared to the other descriptors.

### CUReT

The Columbia-Utrecht Reflectance and Texture (CUReT) dataset [21] contains 61 texture types, such as rabbit fur, styrofoam, pebbles, and moss. It exhibits large uniform changes in illumination conditions. We use only the predominantly front-facing images. For training, we use 3 training images and 7 test images per category, chosen randomly. Example images are shown in Fig. 2A. Chance performance on CUReT is 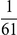.

Our CUReT results are given in Fig. 3B. Performance of grayscale algorithms on CUReT is more variable than AR with several methods performing well for each descriptor type, but members of the Luminance family (*Luminance*, *Luminance* 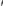, *Luma*, and *Decolorize*) tend to be better than alternatives, with *Luminance* 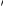 as the top performer for SURF and LBP and *Luminance* being a top performer for SIFT, GB, and LBP. SIFT achieves the best performance, followed by LBP.

### Barnard et al. Dataset

The Barnard et al. dataset [22] contains images of 20 distinct objects. Each object is photographed in 11 different illumination conditions while the pose of the object is simultaneously varied (see Fig. 2B). We chose this dataset because it is the only object dataset that exhibits a variety of systematic changes in lighting color, which we hypothesized would influence many of the grayscale representations. We train on 2 images per object and test on the remaining 9. Chance accuracy is 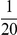.

Two or more methods work well for each descriptor type, but some of them are consistent across descriptors. *Intensity* 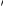 and *Gleam* work well for GB, LBP, and SURF. *Intensity* and *Luminance* perform best for SIFT. Because it is rotation invariant, SURF achieves greater accuracy compared to the other descriptors.

### Caltech 101

The popular Caltech 101 dataset [23] consists of images found using Google image search from 101 object categories, with at least 31 images in each. As shown in Fig. 2C, Caltech 101 has a large amount of interclass variability. We adopt the standard Caltech 101 evaluation scheme. We train on 15 randomly chosen images per category and test on 30 other randomly chosen images per category, unless there are fewer than 30 images available in which case all of the remaining images are used. Chance performance on Caltech 101 is 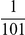.

Our Caltech 101 results are provided in Fig. 3D. Several methods work well, but only *Gleam* performs well for all four descriptors. *Intensity* 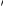 also works well for SIFT, SURF, and LBP. While the choice of grayscale algorithm is significant for Caltech 101, it has a less dramatic effect compared to the other datasets. This is likely because Caltech 101 exhibits less brightness variation and we use a comparatively larger training set. SIFT substantially exceeds the performance of the other descriptors.

The greatest mean per-class accuracy on Caltech 101 is *Luster* 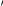, which achieved 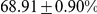 accuracy. For comparison [25], achieved 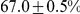 with grayscale SIFT descriptors that had been sparse coded in a hierarchical spatial pyramid matching system.

### Combined Analysis

**Figure 4:**
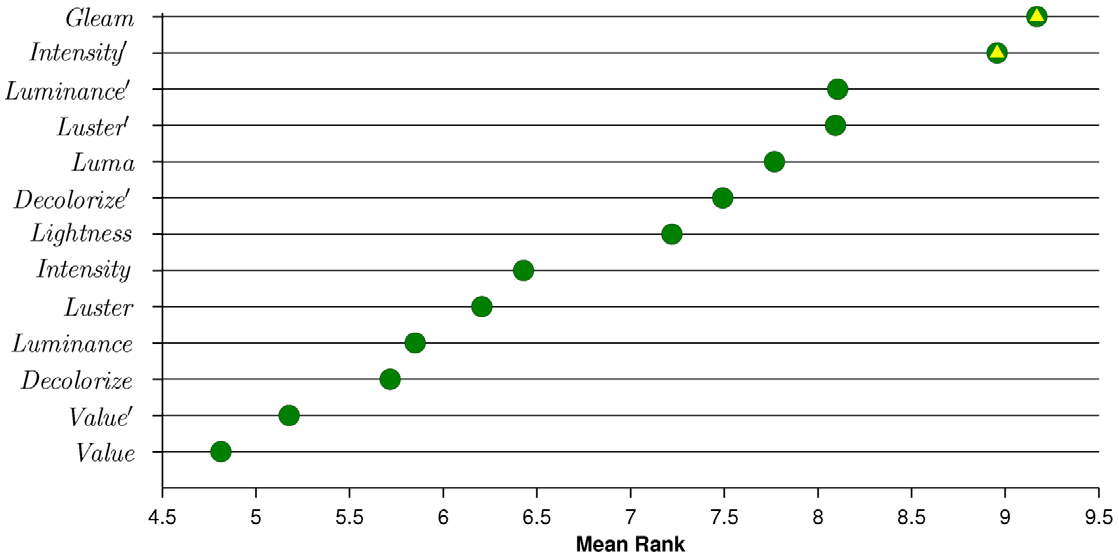
Mean rank results across datasets and descriptor types. The 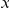-axis is the mean rank for a particular grayscale method when the results are combined across the datasets and descriptor types. *Gleam* and *Intensity* 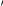 exhibit the greatest rank and most robust performance.

The mean rank performance of each grayscale algorithm marginalized over the datasets and descriptor types is shown in Fig. 4. The simplest methods perform best, with *Gleam* achieving the greatest rank, but it is not significantly different from *Intensity* 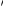. *Value* performs most poorly. Methods incorporating gamma correction are generally superior to their counterparts that omit it, e.g., *Intensity* compared to *Intensity* 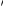 (recall that *Gleam*, *Luma*, and *Lightness* have forms of gamma correction built into them).

**Figure 5:**
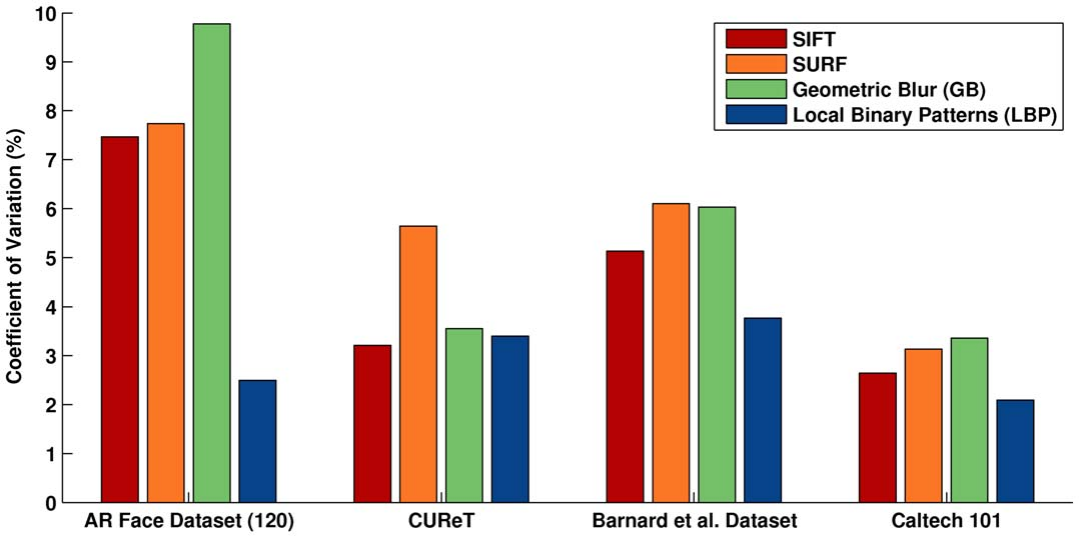
Coefficient of variation for each descriptor type and dataset. The 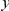-axis is the coefficient of variation for the accuracy of each descriptor type computed across all of the grayscale methods. All of the methods are sensitive to the choice of grayscale algorithm, but LBP is the least sensitive in general. The choice of grayscale algorithm mattered the least for Caltech 101 and the most for the AR Face Dataset.

Our results indicate that each descriptor type is sensitive to the choice of grayscale algorithm. To analyze magnitude of this effect, we computed the coefficient of variation (CV) of each method's performance across grayscale algorithms. These results are shown in Fig. 5. In general, LBP is the least sensitive to the choice of grayscale algorithm, with the only exception being CUReT. For all of the descriptors the choice of grayscale algorithm mattered the least for Caltech 101, probably because of the greater number of training instances and lack of illumination variability.

## Discussion

Our objective was to determine if the method used to convert from color-to-grayscale matters, and we can definitively say that it does influence performance. For all datasets there was a significant gap between the top performing and worst performing methods. Our results indicate that the method used to convert to grayscale should be clearly described in all publications, which is not always the case in image recognition.

For object and face recognition, *Gleam* is almost always the top performer. For texture recognition, *Luminance* 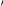 and *Luminance* are good choices. Although color descriptors are sometimes extracted in the HSV colorspace, our results suggest replacing *Value* with *Gleam* is advisable.

In general, we observed little benefit from using a method based on human brightness perception. The only potential exception was textures. Emulating the way humans perceive certain colors as brighter than others appears to be of limited benefit for grayscale image recognition. However, methods that incorporate a form of gamma correction (e.g., *Lightness*, *Gleam*, *Luma*, *Luster* 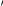, etc.) usually perform better than purely linear methods such as *Intensity* and *Luminance*.

Developing a pre-processing algorithm specifically designed for edge-based and gradient-based descriptors is an interesting future direction. One way to achieve this is to learn a transformation from color-to-grayscale that is robust to changes in brightness, perhaps by allowing the gamma value to vary per color channel, e.g.,
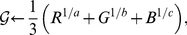
(9)where 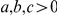 are learned parameters. There is no reason to assume that the single value used in the standard gamma correction function is ideal for recognition. Alternatively, it may be advisable for the transformation weights to vary depending on the global or local statistics of each particular image. In both cases it is challenging to optimize the weights explicitly for recognition since doing so would require re-extracting descriptors. As long as the number of parameters remains relatively small, they could feasibly be optimized per dataset using cross-validation or a meta-heuristic, e.g., genetic algorithms or hill climbing. An alternative is to learn a mapping from color images to descriptors directly. There has been some success with this approach [26], [27], but it has not been widely adopted because these learned transformations tend to be considerably slower than engineered methods (e.g., SIFT) when a comparable descriptor dimensionality is used.

In this paper we asked the question, “Does the method used to convert to grayscale matter in image recognition?” and we have shown that it does significantly influence performance, even when using robust descriptors. The choice made the largest impact for datasets in which only a limited amount of training data was used and illumination conditions were highly variable. We were successful in identifying a method that was consistently superior for face and object recognition. Similarly, for the problem of texture recognition, a pair of top performers emerged. It is now incumbent upon researchers in the computer vision community to report the conversion method they use in each paper, as this seemingly innocuous choice can significantly influence results.
